# What have we learned from 15  years of research on cross-situational word learning? A focused review

**DOI:** 10.3389/fpsyg.2023.1175272

**Published:** 2023-07-04

**Authors:** Tanja C. Roembke, Matilde E. Simonetti, Iring Koch, Andrea M. Philipp

**Affiliations:** Chair of Cognitive and Experimental Psychology, Institute of Psychology, RWTH Aachen University, Aachen, Germany

**Keywords:** cross-situational word learning, cross-situational statistical learning, word learning, language acquisition, review, referential ambiguity

## Abstract

In 2007 and 2008, Yu and Smith published their seminal studies on cross-situational word learning (CSWL) in adults and infants, showing that word-object-mappings can be acquired from distributed statistics despite in-the-moment uncertainty. Since then, the CSWL paradigm has been used extensively to better understand (statistical) word learning in different language learners and under different learning conditions. The goal of this review is to provide an entry-level overview of findings and themes that have emerged in 15 years of research on CSWL across three topic areas (mechanisms of CSWL, CSWL across different learner and task characteristics) and to highlight the questions that remain to be answered.

## Introduction

As described by Quine (1960), any linguistic learning situation by itself may be ambiguous because a novel word form can map on many different present referents or even absent, abstract entities: When you hear the novel word GAVAGAI, you do not know if it refers to the bunny you see, its ears or the grass it is hopping on. However, as pointed out by Yu and Smith (2007; but see also Siskind, 1996; Akhtar and Montague, 1999), the so-called problem of referential ambiguity only exists if a single situation is considered by itself; across many time points, enough distributed statistics may be available to allow for the learning of the correct word-meaning-mappings under the assumption that a word and its meaning are more likely to co-occur than a word and other potential referents. That is, as you hear the word GAVAGAI over and over again, you may be able to extract what it means by learning what other objects or context it co-occurs with. Yu and Smith (2007) tested this hypothesis in adults and in 12- and 14-month-old infants (Smith and Yu, 2008), and found that all age groups were able to acquire the word-object-mappings based on co-occurrence statistics only. This type of word learning has since been termed cross-situational word learning (CSWL).^1^

**Figure 1:**
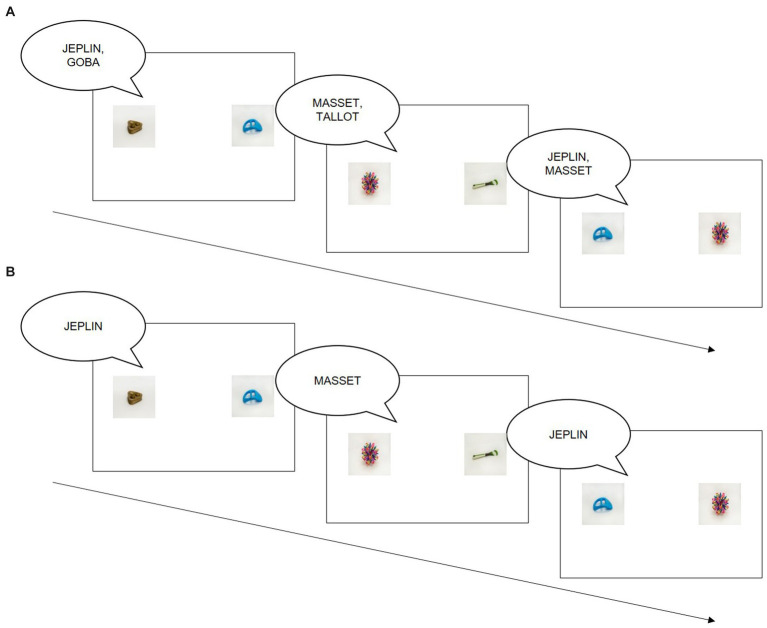
Examples of the two most common variants of the cross-situational word learning paradigms. In Variant 1 **(A)**, participants hear two novel words and see two novel objects on each trial in a passive learning phase (e.g., Yu and Smith, 2007). There is a separate testing phase where participants only hear one word and have to select the correct referent on each trial (similar to Variant 2). In Variant 2 **(B)**, participants only hear one word and see two objects in a two-alternative forced choice trial (e.g., Roembke and McMurray, 2016). There is no separate testing phase. In both variants, it is unclear which of the two objects maps onto the blue object in Trial 1. In Trial 3, however, the word JEPLIN is presented with the target object and a different competitor than in Trial 1. At this point, a participant could know that JEPLIN maps onto the blue object. Many features can be manipulated in this paradigm (e.g., modality of the word, number of presented competitors; number of presented words).

In a typical CSWL experiment, participants hear one or more novel words and see several novel objects. While any trial by itself is ambiguous, the correct word-object-mappings can be learned by tracking word-object-co-occurrence over time. While a word and its target object are always present on the same trial, foil objects differ across trials. Participants are typically instructed that their task is to learn which object each word maps onto, but are not told that co-occurrence indicates a correct mapping (Yu and Smith, 2007). In some variations of the paradigm, participants complete a separate (passive) learning and (active) testing phase (e.g., Yu and Smith, 2007; Escudero et al., 2016c; Poepsel and Weiss, 2016; Figure 1A); in others, they always have to select an object on each trial (e.g., Trueswell et al., 2013; Dautriche and Chemla, 2014; Roembke and McMurray, 2016; Figure 1B). Final accuracy is typically assessed right after learning, though there is a small number of studies that also tested retention at a later time point (Vlach and Sandhofer, 2014; Vlach and DeBrock, 2019; Walker et al., 2020; McGregor et al., 2022). In each variation of the CSWL paradigm, importantly, participants never receive feedback as to whether they selected the correct referent or not.

At the time of writing this review, Yu’s and Smith’s papers were cited 695 (2007) and 939^2^ (2008) times, respectively, reflecting the wide interest in CSWL. The standard CSWL paradigm has been combined with other methods, such as eye-tracking (e.g., Fitneva and Christiansen, 2011, 2017; Yu and Smith, 2011; Yu et al., 2012; Trueswell et al., 2013; Roembke and McMurray, 2016), event-related potentials (e.g., Angwin et al., 2022; Mangardich and Sabbagh, 2022) or fMRI (Berens et al., 2018), and adapted to allow for more detailed trial-by-trial analyses of behavior (e.g., Suanda and Namy, 2012; Trueswell et al., 2013; Dautriche and Chemla, 2014; Roembke and McMurray, 2016, 2021), where accuracy on a current trial is predicted by characteristics of preceding ones. CSWL has also inspired a number of computational models of word learning (e.g., Frank et al., 2009; Fazly et al., 2010; Vogt, 2012; Yu and Smith, 2012; Yurovsky and Frank, 2015; Blythe et al., 2016; Kachergis et al., 2017; Stevens et al., 2017; Bhat et al., 2022).

Now, approximately 15 years after the publication of Yu’s and Smith’s seminal papers, it is our goal to take stock of the literature, to identify the themes that have emerged in the research on CSWL and to highlight the questions that remain to be answered. We will also highlight differences between results from CSWL studies and unambiguous word learning studies (i.e., paradigms without referential ambiguity, such as the explicit pairing of a word with its meaning) when appropriate. The goal of this focused review is not to provide a comprehensive overview of the research that has been conducted on CSWL—this would be beyond the scope of this paper—but rather to provide a broad entry-level road map to past, present and future research on CSWL. As such, we hope that this overview will be helpful for both researchers that have already conducted research on CSWL but also those that are new to the field. We will review research within three sections: (1) Mechanisms of CSWL, (2) CSWL across different learner characteristics, and (3) CSWL across different task characteristics.

### Mechanisms of cross-situational word learning

**Figure 2:**
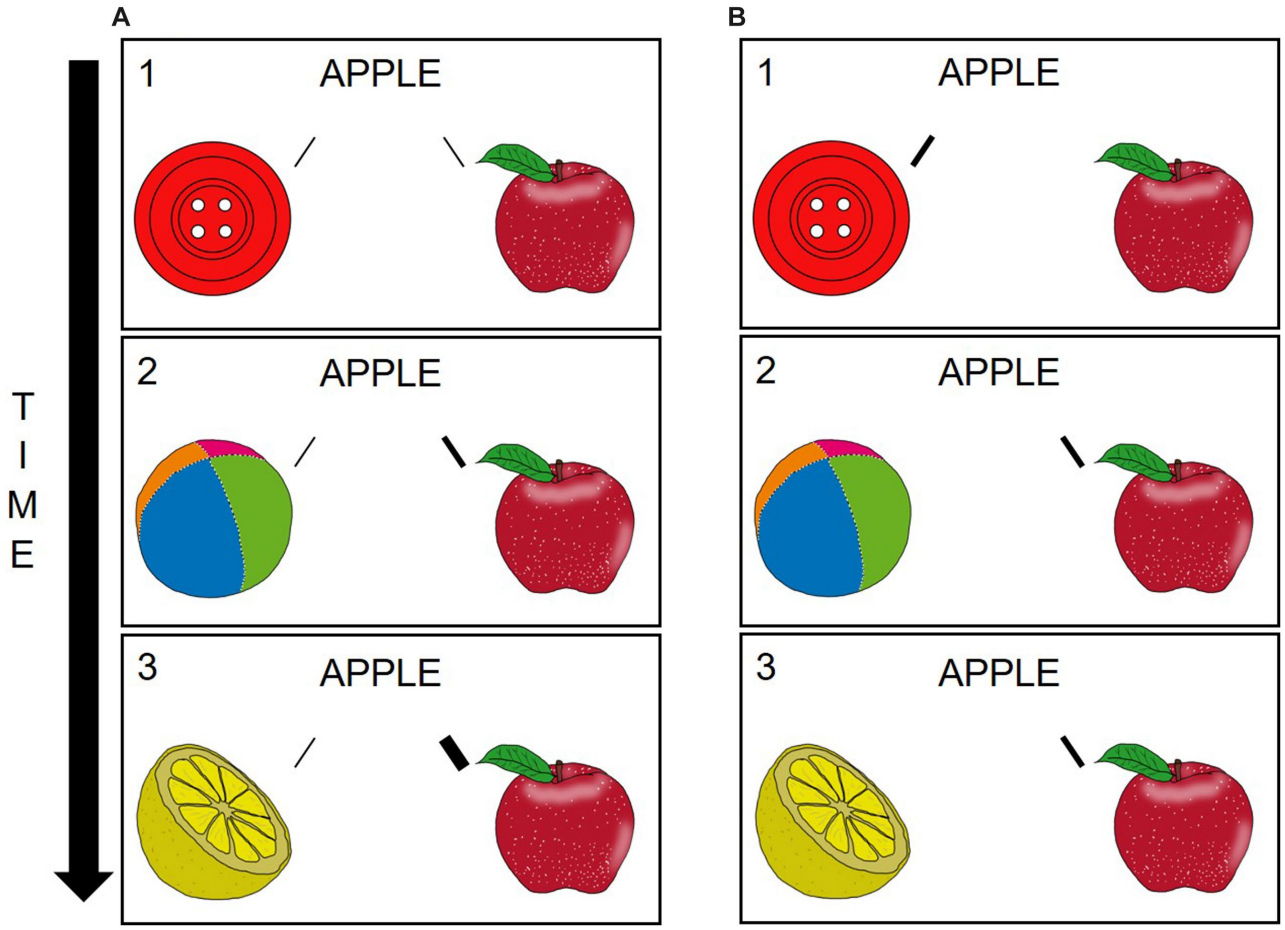
Overview of the two most influential accounts of cross-situational word learning (CSWL). In a gradual associative account **(A)**, associations are built with all possible referents (Trial 1). As the correct word-object-mapping co-occurs more frequently than any other word-competitor-pairing, their association becomes strongest (Trial 3). In contrast, in a propose-but-verify (hypothesis-testing) account, people form one hypothesis about each word-object-mapping **(B)**. In this example, the wrong hypothesis is “proposed” on Trial 1, which is then verified and revised on subsequent trials (Trials 2–3). Images are retrieved from the MultiPic database (Duñabeitia et al., 2018).

In Yu and Smith’s (2007) original study, CSWL was considered a type of statistical learning with an underlying associative, domain-general mechanism: Across trials, associations between co-occurring words and referents are gradually strengthened, whereas associations between non-co-occurring items remain weak (Figure 2A; Yu and Smith, 2007, 2012; Kachergis et al., 2012). One core assumption of this gradual associative mechanism is that people can maintain multiple hypotheses (e.g., between a word and its eventual target object as well as competitors) simultaneously. During learning, different mappings may then compete with each other, both within the same trial as well as across trials (Fazly et al., 2010; Yurovsky et al., 2013; Benitez et al., 2016). Another assumption of the gradual associative mechanism is that at least some of the learning is implicit with CSWL being a form of statistical learning (Frost et al., 2019; Weiss et al., 2020). In Yu and Smith’s (2007) original study, for example, awareness was not necessary to acquire the word-object-mappings.

As an alternative, the propose-but-verify (hypothesis-testing) account was put forward (Figure 2B; Medina et al., 2011; Trueswell et al., 2013; Woodard et al., 2016). In this account, people are thought to only maintain one hypothesis about each word’s meaning, which is verified and, if needed, revised. As a result, in this account, CSWL is more adequately described as a type of fast-mapping procedure than as a gradual associative one (Trueswell et al., 2013). In propose-but-verify, learning is generally thought to be more dependent on participants’ awareness (i.e., to be explicit).

Several studies have tested the conflicting predictions that people can maintain multiple hypotheses per word (gradual associative account) or not (propose-but-verify/hypothesis-testing account). Evidence for propose-but-verify originally came from trial-by-trial autocorrelation analyses showing that participants were at chance on a current trial if they had not picked the correct referent previously (Trueswell et al., 2013): If only one hypothesis is maintained, learning must be at chance if the proposed target is not available for selection on a current trial and a new target hypothesis has to be formed. However, since then, it has been shown across several different paradigm variants that multiple hypotheses can be maintained in parallel (e.g., Dautriche and Chemla, 2014; Yurovsky et al., 2014; Roembke and McMurray, 2016). For example, Yurovsky et al. (2014) first trained participants on a set of word-object-mappings in a CSWL task. Subsequently, they exposed participants to a set of new word-object-mappings as well as ones from the first training that had not been learned. Participants were found to be better at acquiring the words that they had received training on before, suggesting that they had retained some partial knowledge from the previous training besides having been at chance performance for these words then. Similarly, Roembke and McMurray (2016) observed that participants were more likely to look at object competitors that had been more frequently paired with the word than a baseline object, even as they clicked on the correct target object. This looking behavior is consistent with the maintenance and parallel in-the-moment activation of multiple mappings per word. Given that multiple hypotheses are tracked per word, one question that remains to be answered is how incorrect meanings may be unlearned to facilitate the activation of the correct referent (McMurray et al., 2012).

More recently, instead of seeing the two accounts as opposing, it was suggested that they may represent two distinct learning systems that work in parallel during CSWL. Here, the core of learning is associative and gradual, but the formation of explicit hypotheses and attention allocation can impact how associations are formed (McMurray et al., 2012; Yurovsky and Frank, 2015; Roembke and McMurray, 2016). Consistent with a mixed account, for example, Kachergis et al. (2014) found that adults were able to acquire word-object-mappings via CSWL even in the absence of an explicit effort to learn; at the same time, acquisition was also superior under explicit study instructions. The relative reliance on different learning mechanisms (e.g., gradual accumulation of statistics versus propose-but-verify/hypothesis-testing) may vary with a number of factors, including the degree of ambiguity, the number of unfamiliar words, familiarity of visual referents (e.g., whether they can be easily described or not), context (e.g., cover task or not) and task (e.g., task instructions, time pressure to respond) but also people’s beliefs and confidence during learning (e.g., Wang and Mintz, 2018; Wang, 2020; Dautriche et al., 2021).

In addition, CSWL mechanisms may also differ across development: In younger children, there is evidence that only one hypothesis is maintained per word (Woodard et al., 2016; Aravind et al., 2018). At the same time, it has also been suggested that implicit, associative learning is actually *more* common in young children than adults (Ramscar et al., 2013). Evidence for a qualitative change in CSWL mechanism across development also comes from a study by Fitneva and Christiansen (2017). They manipulated to what extent three age groups (4-year-olds, 10-year-olds and young adults) were exposed to more initially incorrect (mismatched) word-object-mappings or not. Surprisingly, it was found that while 4-year-olds’ CSWL benefitted from initial accuracy, young adults’ learning was best if they were exposed to an incorrect mapping at first (no learning difference due to initial accuracy/inaccuracy was observed for 10-year-olds). Moreover, the learning benefit due to accurate (4-year-olds) and inaccurate (young adults) initial mappings was not specific to the manipulated items but applied to the whole set of to-be-learned words. While the exact mechanism behind the initial inaccuracy/accuracy benefit is unclear, it suggests an important role of memory and attention during CSWL (Fitneva and Christiansen, 2017).

Consistent with this interpretation, recent work by Vlach and DeBrock (2017, 2019) suggests that memory may be the best predictor of 2- to 6-year-olds’ CSWL performance (more so than their age or vocabulary size). At the same time, in adults, neither working memory nor phonological short-term memory predicts overall CSWL (Walker et al., 2020). Yet, our understanding of CSWL across development is currently limited by not knowing when statistical word learning is most common: Do children learn words cross-situationally when they are infants and more limited in their ability to actively shape their environment (Kachergis et al., 2012; Smith et al., 2014)? Or is it the most common learning mechanism of new words in older children and adults who acquire most of their vocabulary by reading (Nagy et al., 1985, 1987)? Answering these questions may give us insights into the type of mechanisms that are most likely to be engaged during CSWL at different ages.

As mentioned previously, the experimental research on CSWL has also inspired a number of computational models, which have helped test the plausibility of CSWL in general (e.g., Blythe et al., 2010; Vogt, 2012) and different CSWL mechanisms more specifically (e.g., Frank et al., 2009; Fazly et al., 2010; Yu and Smith, 2012; Yurovsky and Frank, 2015; Kachergis et al., 2017; Stevens et al., 2017; Bhat et al., 2022). In fact, Bhat et al. (2022) recently identified 19 different models that range in their theoretical founding (gradual associative, propose-but-verify/hypothesis-testing or mixed), the input they take (e.g., symbolic stimuli) as well as their computational formalism (e.g., connectionist or Bayesian; see Bhat et al., 2022 for a detailed review of the different models), with the associative models by Kachergis et al. (2012, 2013, 2017) being highlighted as very successful at explaining data from several studies. However, many of these models (including the ones by Kachergis et al., 2012, 2013, 2017) fail to consider children’s developing cognitive abilities (Vlach and DeBrock, 2019). One recent exception is the WOLVES, a dynamics field theory model by Bhat et al. (2022), which was able to model infants’, toddlers’ and adults’ (looking) behavior across development.

To summarize, the two original competing accounts—gradual associative/statistical and propose-but-verify/hypothesis-testing—have been very helpful in framing research on CSWL, as they provided specific testable hypotheses. The resulting data suggest that CSWL is most accurately described by a mixed account that can incorporate findings that are more in line with gradual or statistical learning as well as ones in line with propose-but-verify (hypothesis-testing). Moving forward, taking into account the developmental time line of this learning represents an important new step. Moreover, most research on CSWL has been concerned with the mechanisms of how initial word-object-mappings are established but less so with how newly acquired meanings are retained (as reflected in the small number of studies that test retention of word meanings, e.g., Walker et al., 2020; McGregor et al., 2022) and integrated in the broader lexicon.

### Cross-situational word learning across different learner characteristics

The plausibility of CSWL as a form of word learning is limited if it can only be shown in (neurotypical) adults. As such, Smith and Yu (2008) provided a litmus test by adapting the original adult paradigm that used accuracy as the dependent variable to an eye-tracking paradigm to be used with infants. As highlighted previously, they showed that 12- and 14-month-old infants were able to learn word-objects-mappings from cross-situational statistics after a short exposure. Since then, CSWL has been observed in children of different age groups (e.g., Smith and Yu, 2008; Scott and Fisher, 2012; Suanda et al., 2014; Bunce and Scott, 2017; Vlach and DeBrock, 2017; Roembke et al., 2018; Benitez et al., 2020; Crespo and Kaushanskaya, 2021; Mangardich and Sabbagh, 2022), children with developmental language disorder (DLD; Ahufinger et al., 2021; McGregor et al., 2022), children with autism (Venker, 2019; Hartley et al., 2020) and late talking children (Cheung et al., 2022) as well as older adults (Bulgarelli et al., 2021), adults with hippocampal amnesia (Warren et al., 2020) and aphasia (Peñaloza et al., 2017). CSWL was also effective when learning words in a second language (Hu, 2017; Tuninetti et al., 2020). For an exception in which no evidence for learning was found when testing CSWL in an isolated community in Papua New Guinea, the unfamiliarity with laboratory-based experiments of these participants is a likely explanation (Mulak et al., 2021). Overall, it is clear that language learners with different cognitive profiles can acquire word-object-mappings via distributed statistics as in CSWL, even as learning is typically lower in a CSWL than in a unambiguous word learning task where word and meaning are explicitly paired (Mulak et al., 2019). Nevertheless, there are (at least) two methodological limitations to many of these studies: (1) they often only assess learning right after exposure, leaving unclear how long-lasting the acquired representations are; and (2) because the goal of these studies was to show if CSWL was possible at all, the to-be-learned statistical relationships are very simple (i.e., low number of words to be learned; low referential ambiguity within each trial); thus, it is an open question whether CSWL would also be possible if more complex statistical relationships had to be tracked.

While the previous studies on different learners found performance above chance at the group level, there is often considerable variability at the individual level, sometimes to the extent that it is not clear whether successful CSWL is universal enough on a person to person basis to contribute meaningfully to an all-encompassing explanation of word learning. In young children, for example, it has been questioned whether CSWL is robust and whether its resulting representations are long-lasting (Vlach and Johnson, 2013; Aravind et al., 2018; Vlach and DeBrock, 2019). Similarly, adult participants with hippocampal amnesia (Warren et al., 2020) and aphasia (Peñaloza et al., 2017) were able to acquire words cross-situationally; however, their learning was at a slower rate than age-matched control groups. In addition, while learning was above chance at a group level/in some participants^3^, this was not true for a substantial subset of the samples. For example, seven out of 16 participants with aphasia performed at chance in a simple CSWL task (Peñaloza et al., 2017)—the result that a majority did learn is impressive, but still calls into question why CSWL was not accessible to the non-learners.

Investigations into the impact of individual differences on CSWL are limited in number. Consistent with the idea that attention is an important determinant of whether statistical co-occurrences are encoded, differences in selective sustained attention can explain some variability in CSWL, with strong learners having fewer fixations with longer durations than weaker learners (Yu and Smith, 2011; Yu et al., 2012; Smith and Yu, 2013), which may reduce the number of incorrect associations that are encoded (Bhat et al., 2022). In addition, it has been hypothesized that a person’s language skills drive CSWL ability (rather than the other way around; Vlach and DeBrock, 2017). Data from Scott and Fisher (2012) were interpreted to be consistent with this hypothesis, as they found that toddlers with larger vocabularies also performed better on more difficult CSWL tasks than children with smaller vocabularies. Investigating individual differences in CSWL ability is not straightforward; to our knowledge, there is currently no data on whether a typical CSWL task is a reliable measure of an individual’s statistical word learning ability (versus group-level ability; see the general statistical learning literature on a discussion of this; e.g., Siegelman et al., 2017). In a typical CSWL experiment, participants are naïve to the existence of co-occurrence statistics; this would no longer be the case if they repeatedly participated in CSWL tasks.

Furthermore, CSWL does not only differ between individuals within a specific group but also between groups. Such differences in CSWL performance across groups are multi-faceted: For example, children with DLD (McGregor et al., 2022) were found to score lower than age-matched controls when learning words cross-situationally. Interestingly, for children with DLD, a performance gap emerged early on during the CSWL task, while later learning occurred at a similar rate as in the controls (McGregor et al., 2022). This suggests that initial encoding during CSWL may be the bottleneck, whereas the actual mapping of words onto meanings based on co-occurrence is not affected in children with DLD. At the same time, late-talking children learned words at the same rate as controls during CSWL training, but were worse at retention (Cheung et al., 2022). Finally, children with autism performed similarly to vocabulary-matched controls in a CSWL task, but were slower to pick the correct referents (Hartley et al., 2020). It is possible that some of these CSWL learning differences across groups contribute to observed language delays (e.g., smaller overall vocabularies), though a causal relationship is currently not well-established. Further research should also explore whether CSWL ability is an appropriate intervention target to mitigate a language delay (c.f., Alt et al., 2014).

Group differences are also observed between monolinguals and bilinguals: Consistent with results from unambiguous word learning paradigms, bilinguals outperformed monolinguals in some CSWL studies (Escudero et al., 2016c; Poepsel and Weiss, 2016; Crespo et al., 2023) but not all (Benitez et al., 2016; Crespo and Kaushanskaya, 2021), suggesting that differences in language learning history may impact how easily words are acquired statistically. However, the circumstances under which bilinguals outperform monolinguals are currently not well understood, with some suggesting that a bilingual advantage may only exist when more complex word-object mappings are acquired (Poepsel and Weiss, 2016) or when words include multiple sources of phonological variability (Crespo et al., 2023). Given these mixed results, there is a need for more research on how bilingualism may affect statistical word learning. In this context, it is also important to consider how more complex mappings between words and objects (e.g., one word maps onto several meanings), which tend to be more common for bilinguals, can be acquired via CSWL (Poepsel and Weiss, 2016).

In summary, language learners with different cognitive profiles can acquire words cross-situationally. However, at this point, these studies often represent an existence proof (i.e., are the mappings learned at all?), and are thus limited to relatively simple learning situations. In addition, most of the studies are conducted with English-speaking participants (but see exceptions in subsequent section), leaving unclear to what extent statistical word learning may be common in other languages and cultures (but see Mulak et al. (2021) for an exception). While there is some suggestive evidence that a deficit in CSWL ability may contribute to language delay in some learners, the underlying mechanisms are not well-understood. As the relationship between individual differences and CSWL has been mostly investigated in young children, it is currently unclear to what extent it is predicted by individual differences in vocabulary or memory in older children and adults. Thus, there currently is no good understanding of what individual differences predict CSWL performance.

### Cross-situational word learning across different task characteristics

Another theme that has emerged in the research on CSWL is what characteristics within a learning context impact how easily a word is acquired. We will first review research on the impact of trial-by-trial characteristics on CSWL followed by a closer look at the impact of stimulus characteristics.

#### Trial-by-trial characteristics

Different trial-by-trial characteristics like the amount of referential ambiguity (number of objects on the screen), number of to-be-learned words, how often each word is repeated, and the complexity of the word-object-mappings impact CSWL: Observed learning rate will be highest if there are only two visual referents on each screen and a small number of to-be learned one-to-one-mappings (i.e., each word maps onto one referent only) that are repeated often (e.g., Yu and Smith, 2007; Poepsel and Weiss, 2016; Roembke and McMurray, 2016). Some studies have combined the learning of cross-situational statistics with other cues, such as morphological (Finley, 2022) or social ones (Frank et al., 2013; MacDonald et al., 2017); these can facilitate how easy it is to learn or what type of information is encoded.

In a typical CSWL experiment, trials are randomized completely or pseudo-randomized to avoid direct repetitions of the same word within a larger block (e.g., Roembke and McMurray, 2016; Hartley et al., 2020; Escudero et al., 2022; Yip, 2022). Both children and adults are more likely to be correct on a current trial if they completed a trial with the same word more recently, suggesting recency facilitating retrieval of previous learning episodes (Roembke and McMurray, 2016; Roembke et al., 2018). Research has found that children perform similarly for when CSWL trials are presented in massed order (i.e., no or few interleaved trials between repetitions) and interleaved order (i.e., many interleaved trials between repetitions) (Smith and Yu, 2013; Vlach and Johnson, 2013). At the same time, however, adults substantially benefited from massing (Benitez et al., 2020). In infants, massing may hurt CSWL, as it leads to the visual habituation to the referents which in turn then hurts their encoding (Smith and Yu, 2013). Adults’ cross-situational learning is best if they can decide which objects are presented to them next, thus optimizing the order in which they receive information (Kachergis et al., 2013). Temporal order has also been found to matter on a smaller time-scale: within-each trial. Apfelbaum and McMurray (2017) observed that whether words and objects were presented synchronously or not during CSWL impacted how mappings were linked, as participants were more likely to form spurious incorrect associations with competitor objects when auditory and visual stimuli were present at the same time.

Another common feature of CSWL experiments is that each word is presented an equal number of times. However, it has been argued that such a uniform frequency distribution is not representative of the real world (Blythe et al., 2010; Vogt, 2012; Hendrickson and Perfors, 2019), where some words are much more likely to be encountered than others (Zipfian distribution). Thus, a recent study by Hendrickson and Perfors (2019) compared CSWL when both words and meaning were either distributed uniformly or not. Surprisingly, it was found that participants learned better in the non-uniform, Zipfian distribution than the uniform one, likely because they were able to use high-frequency words to disambiguate the meaning of low-frequency ones (Hendrickson and Perfors, 2019).

#### Stimulus characteristics

In a typical CSWL study, words are newly generated non-words that have minimal overlap, follow legal phonotactics (e.g., FEP, DAX or GOBA for speakers of English) and are presented in spoken form by a native speaker (e.g., Fitneva and Christiansen, 2011; Vlach and Sandhofer, 2014; Roembke and McMurray, 2016). In addition, the vast majority of CSWL studies was conducted with English-speaking participants, thus implementing and investigating word characteristics that are common in alphabetic, Western languages (e.g., CVCV structure of to-be-learned words; c.f. Yip, 2022).

Work with minimal word pairs (e.g., BON/TON or DEET/DIT; Escudero et al., 2016b) showed that English-speaking adults and even infants encode fine phonological consonant and vowel detail when learning words via CSWL (Escudero et al., 2016a,b; Mulak et al., 2019). Yip (2022) also found that native speakers of Cantonese Chinese encoded phonological detail during CSWL but that tonal information was more critical to learning than other types of phonological information. It is not clear to what extent phonological familiarity and variability of words can facilitate CSWL as a downstream benefit of easier word form encoding (as has been observed in unambiguous word learning studies; e.g., Rost and McMurray, 2010): While it was found to be easier to map words than non-linguistic beeps onto objects for adults (but not for children; Roembke et al., 2018), phonotactic legality of words only had a small impact on CSWL, if at all (Dal Ben et al., 2022). Similarly, no learning benefit was observed when words were presented via multiple speakers versus a single one (Crespo and Kaushanskaya, 2021). Nevertheless, words are more easily acquired cross-situationally if they are presented visually rather than auditorily, which is consistent with results from other unambiguous word learning paradigms (Escudero et al., 2022).

There is only very little work on how variability in the visual referents may impact cross-situational word learning. One exception is a study by Wang (2020) which reported that participants were more likely to use implicit learning mechanisms if objects could not be described easily. In addition, Monaghan et al. (2015) found that CSWL was robust even when objects moved on the screen (also see Walker et al., 2020; Rebuschat et al., 2021). It is currently not known whether shapes that are more closely related to already existing semantic representations are easier/harder to encode and/or how variability in the visual exemplars may impact how word-object-mappings are acquired statistically.

CSWL is mostly tested with non-words and novel objects as referents; thus, the non-words are created to mimic concrete basic-level nouns. However, there are also studies that explore how different types of words are acquired via CSWL. Recent investigations suggest that learning subordinate-level word meanings (e.g., the word DALMATIAN) may be more difficult during CSWL than the learning of basic-level ones (e.g., DOG; Wang and Trueswell, 2019, 2022). Scott and Fisher (2012) showed that toddlers could learn novel verbs with the help of distributed statistics. Similarly, Monaghan et al. (2015) found that nouns and verbs embedded in short “sentences” can be learned simultaneously cross-situationally in the absence of syntactic cues. In a new variant of the CSWL paradigm (Rebuschat et al., 2021), participants are exposed to multi-word utterances and complex scenes. Thus, participants’ task is to not only learn word-object-mappings but to extract both grammar and vocabulary from cross-situational statistics without receiving feedback. Despite the increased complexity, participants were able to learn the sentence-to-scene correspondences (Rebuschat et al., 2021). These findings have since been replicated and extended by Walker et al. (2020) who also tested retention after a 24 h delay, observing performance improvements for some word types after consolidation. While these studies still do not contain the complexity of natural language acquisition, they represent an intriguing next step in showing that CSWL is not an artificial phenomenon limited to a small set of word types under low referential ambiguity (Walker et al., 2020; Rebuschat et al., 2021).

#### Summary

To summarize, many different factors impact how easy it is to acquire a word during CSWL. The variables investigated range from the methodologically important (e.g., number of objects on screen/referential ambiguity which impacts chance level) to the potentially more theoretically interesting ones (e.g., variability in word exemplars). At the same time, it is surprising to see that some factors that have been robustly found to influence word learning in other paradigms (e.g., massing/interleaving for children, phonotactic familiarity) are potentially less important in CSWL. Our understanding of CSWL will further improve as future studies move beyond investigating the acquisition of isolated nouns of English-sounding words (Monaghan et al., 2015; Walker et al., 2020; Rebuschat et al., 2021; Yip, 2022).

## Conclusion

The basic phenomenon of CSWL is well-established across a high number of populations, stimuli and learning conditions. It is exciting to see how far the field has come since Yu’s and Smith’s original seminal publications in 2007 and 2008, testing different mechanisms of CSWL and adding complexity to the CSWL paradigm at different levels of the learning process. However, there also remain questions that deserve more attention in the next 15 years of research (see Textbox 1 for a non-exhaustive list).

**TEXTBOX 1: Overview of some open questions on cross-situational word learning (CSWL).**

1. When (i.e., at what age) is CSWL most common? How does CSWL differ across different age groups?
2. How does CSWL relate to other forms of word learning and word  learning outcomes?
3. Is a deficit in CSWL implicated in language delay? Is CSWL a possible target for word learning interventions?
4. What individual differences predict CSWL performance?
5. To what extent do differences in learning experience, such as bilingualism, impact CSWL?
6. How does CSWL occur for more complex statistical relationships  (e.g., multiple mappings per words)?
7. How are incorrect mappings unlearned as part of CSWL?
8. How does CSWL occur in languages other than English? Is it more  common in some populations/languages than others?
9. How well are words retained that are acquired via CSWL? How quickly are they integrated into the broader lexicon?
10. How does CSWL relate to other types of implicit/statistical learning?
11. What is the role of awareness in CSWL?

## Author contributions

TR drafted the first version of the manuscript. MES, IK, and AMP commented on and edited the manuscript. All authors contributed to the article and approved the submitted version.

## Funding

The research reported in this article was supported by the Deutsche Forschungsgemeinschaft (Grant No. 505754094) awarded to TCR and IK.

## Conflict of interest

The authors declare that the research was conducted in the absence of any commercial or financial relationships that could be construed as a potential conflict of interest.

## Publisher’s note

All claims expressed in this article are solely those of the authors and do not necessarily represent those of their affiliated organizations, or those of the publisher, the editors and the reviewers. Any product that may be evaluated in this article, or claim that may be made by its manufacturer, is not guaranteed or endorsed by the publisher.

## Footnotes

^1^Cross-situational word learning can also be referred to as cross-situational learning, cross-situational statistical learning, statistical word learning or observational word learning. We will use the term cross-situational word learning throughout this manuscript.

^2^These numbers were returned by a Google Scholar (www.google.com/scholar) search on May 12, 2023.

^3^Due to the small number of amnesic participants, no group analyses were conducted by Warren et al. (2020).
